# The impact of health literacy on uterine fibroid awareness, diagnosis, and treatment in the United States: a mini literature review

**DOI:** 10.3389/frph.2024.1335412

**Published:** 2024-03-11

**Authors:** Joyvina Evans

**Affiliations:** Department of Health Management, Howard University, Washington, DC, United States

**Keywords:** health literacy, literacy, uterine fibroids, fibroids, leiomyomas

## Abstract

Limited health literacy is a social health determinant leading to poor health outcomes. General and health literacy correlate and can impact diagnosis and treatment understanding. Limited literacy can lead to women receiving more significant rates of invasive surgical treatment, including hysterectomies. This review explores the impact of health literacy levels on uterine fibroid awareness, diagnosis, and treatment. PubMed, CINAHL, and Academic Search Premier searched articles published between January 1, 2012, and December 31, 2022. The keywords uterine fibroids, fibroids, myomas, leiomyomas, and health literacy were used. A total of four articles were returned. Increased rates of hysterectomies were found among participants with low income and education, as well as those with limited health literacy. Hysterectomies are the removal of the uterus and thus removes a woman's right to have children. While increased efforts are needed to understand the impact of health literacy levels on disparities and inequities in uterine fibroid diagnosis and treatment, there is still a need for targeted patient education and community-based education that ensures patient understanding of the diagnosis and treatment options for uterine fibroids.

## 1 Introduction

Women's understanding of treatment options, including surgery, is critical to making an informed decision. Health literacy is defined as the possession of the knowledge and literacy skills needed to make an informed health-related decision. Health literacy is linked with reading levels and literacy levels. While there is a correlation, they are all fundamentally different. There are many definitions, but at the core of health literacy are the common elements regarding the ability to obtain, understand, and use health information to make informed decisions regarding health and treatment that will significantly impact health outcomes (1). Studies have demonstrated that people with limited health literacy struggle with medication adherence, communicating effectively with health providers, understanding diagnosis and treatment, increased emergency room visits, and an increase in mortality rates (2).

The American Psychological Association reports that numerous examinations of socioeconomic status reveal inequities concerning access to resources and care. Socioeconomic status (SES), including education and income, influences access to health care, with some treatments directly affected by insurance coverage and the cost of the procedure. While health literacy is not explicitly linked to socioeconomic status, some studies report the association between education status and health literacy levels. SES and race are known to impact access to adequate and quality health care, and access to some forms of uterine fibroid treatments is affected by insurance type and insurance status. The inequity of treatment offering and range is a concern, and more studies are needed on diagnosis and treatment access for women with low and limited health literacy levels. Determining patients' health literacy level can illuminate their understanding of their health conditions and lead to more appropriate health decisions and outcomes.

This review explicitly highlights uterine fibroid diagnosis and treatment decisions. Uterine fibroids are non-cancerous (benign) tumors of the uterus. They are also referred to as leiomyomas and myomas, and symptoms include heavy menses, pain, reproductive issues, anemia, and frequent urination (3). There are many treatment options for uterine fibroids, ranging from a “wait and see” approach to surgical treatments. Myomectomies (removal of fibroids) and hysterectomies (removal of the uterus) are more prevalent in African-American women due to more severe symptoms. African American women are two to three times more likely to undergo a hysterectomy than white women and 6.8 times as likely to receive a myomectomy (4). The rates of hysterectomies increase among women of lower socioeconomic status. Findings reveal that women in the United States with lower socioeconomic status tend not to seek care until the later stages of uterine fibroid diagnosis, sometimes resulting in larger tumors and, thus, an increased rate of hysterectomies. When income and education levels were increased, women were more likely to select less invasive treatment options such as myomectomy, uterine artery embolization, or endometrial ablation (3).

Belilovets (5) reported that over 20 million women had undergone a hysterectomy. Hysterectomy is the most performed gynecological surgery. There are alternatives to hysterectomies, such as medication, uterine artery embolization, ablation, and myomectomies. Due to the additional options, women must understand their diagnosis, the indications for treatment, treatment options, and post-surgery care.

## 2 Methods

This review article investigated the available data regarding the association between health literacy and uterine fibroids. PubMed, CINAHL, Academic Search Premier, and Cochrane Databases were used to search the literature for articles published between January 1, 2012, and December 31, 2022. The search used the following keywords: uterine fibroids, fibroids, uterine leiomyomas, myomas, and health literacy. Articles were excluded if they were conducted outside the United States and if uterine fibroids and health literacy were not the primary focus. Additionally, duplicate articles or similar articles by the same authors were excluded. Four articles were reviewed for this narrative assessment, indicating that this review may be early in a research cycle focusing explicitly on the link between health literacy and uterine fibroid awareness and treatment.

## 3 Results

According to the results of a study conducted by Ekpo et al. (6), over 46% of the participants had incorrect knowledge of uterine fibroids and believed that a blood test could diagnose uterine fibroids. This study aimed to assess awareness and knowledge of uterine fibroid symptoms. There was a 14.1% prevalence of inadequate health literacy levels. All participants were African-American women, and approximately 34% had some form of a college education. Most women had common knowledge that uterine fibroids lead to heavy menstrual cycles and can increase the chance of a miscarriage. While this information was determined through the survey of 199 women, there was no significance between health literacy levels and overall fibroid knowledge. The study found that participants who used the internet and those with higher education were more aware of uterine fibroids.

Ghant et al. (7) conducted a research project where women with uterine fibroids were interviewed. The mean age of participants was 43 ± 6.8. Sixty women were included in the project, with approximately 62% of the participants identifying as African American and 25% as Caucasian. The researchers revealed that some women had limited knowledge of uterine fibroids and normal menstruation, which led to treatment delays and a delay in the ability to take action regarding their diagnosis. Many participants reported awareness of the issue but desired to avoid the diagnosis and symptoms.

An anonymous population-based survey was distributed to women in a study designed to assess understanding of hysterectomies and uterine fibroids and included 28 knowledge questions and ten demographic questions. The knowledge questions focused on hysterectomy indications, hysterectomy types, procedures, post-hysterectomy care, and complications (5). A total of 200 surveys were collected. Over 43% of the participants identified as Caucasian, 31% as Hispanic, 11% as Asian, and 10% as African American. Approximately 28.5% of the participants reported less than high school and high school education. The results of the study demonstrated a poor understanding of the hysterectomy procedure. Many participants did not understand uterine fibroids and did not understand different hysterectomy approaches. Eight percent of women answered the question regarding the type of hysterectomy that leads to a greater risk of bladder compromise incorrectly. It is important to note that this question was written at a lower than an eighth-grade level. In contrast, the participants demonstrated the most knowledge of the risk of hysterectomies.

Marsh et al. (8) conducted a population-based survey to determine patient awareness and treatment decisions for patients diagnosed with uterine fibroids. Women were grouped into at-risk (*n* = 300), diagnosed (*n* = 871), and uterine fibroid-related hysterectomy (*n* = 272) categories. Approximately half of the participants in the at-risk group were aware of uterine fibroids. Women with an income over $60,000 had heard of fibroids. Over 60% of women in the at-risk group tried to manage symptoms from uterine fibroids and hoped they would go away. African-American women in the at-risk group were less likely to make an appointment with a provider than White women. In the diagnosed group, 71% received some form of pharmacologic therapy. They were followed by 30% who underwent some form of a procedure. The average age of participants who underwent a hysterectomy was 41, with 11% undergoing a hysterectomy before they turned 35. Of the women who had a hysterectomy, the top reason was a recommendation from a provider (58%), followed by 55% due to pain. One-third of participants with a hysterectomy indicated an interest in a uterus-sparing option.

## 4 Discussion

This review demonstrates the impact of health literacy and knowledge on awareness of uterine fibroids and treatment understanding and selection. Improved health literacy can increase patient and community trust, decrease health disparities, and improve health outcomes. There are studies on health literacy and women's health; however, limited information focuses on health literacy, uterine fibroids, and treatment for uterine fibroids. Limited health literacy levels and lower education were linked to increased hysterectomy rates and lower awareness and understanding rates. While the review presents needed information on health literacy rates on uterine fibroid treatment options, it does have limitations. The main limitation is not conducting a quality assessment of the articles selected for the review. Only articles published in peer-reviewed journals were included. Only studies published in English and the United States were included.

There are many uterine fibroid articles and many articles that focus on health literacy and other health outcomes; however, articles that specifically deal with health literacy “and” uterine fibroids are limited, highlighting the need for more research. The knowledge and understanding deficiencies are tied to health literacy and other factors or determinants of health. Ensuring that providers provide all of the required information and details in an easy-to-understand manner is essential. Health literacy levels cannot be assumed and are not something that a chart review or conversation with the patient can determine. At the same time, there are limited articles that specifically discuss uterine fibroids or hysterectomies; the studies that specifically focus on health outcomes are associated with decreased use of preventive services and higher mortality rates, mainly among the elderly (9). It is essential to determine if the number of women who undergo hysterectomies is aware of all of the treatment options or if they opt for a hysterectomy due to limited knowledge.

It is important to note that mobile health, telehealth, and the increase in digital health technologies may present additional challenges to people with limited literacy. Providers can only assume that some patients have smartphones, computers, or internet capabilities. Beyond this, providers cannot assume that patients know how to work the devices or maneuver through the website or online platform. Smith and Magnani (10) studied the intersection of electronic health and digital health literacy. The study highlights that populations with a high risk of limited health literacy are likely also to have challenges with digital health literacy. For instance, people with poor digital or eHealth literacy suffer from more chronic health conditions.

The review highlights the need for additional patient education. There is a need for patient-centered education as well as community-based education. Targeted education that is culturally appropriate and at needed health literacy levels may increase the likelihood of informed decision-making. Increasing the knowledge and understanding of women diagnosed with uterine fibroids ensures that they feel empowered regarding their decisions and have limited regrets. Studies have shown that using multimedia tools and other patient education improves knowledge of uterine fibroids and anxiety levels (11). Therefore, providers should seek to use additional tools to reach patients, especially those with limited or low health literacy.

## 5 Conclusion

Women with limited health literacy levels and lower education have increased rates of invasive surgical procedures, such as hysterectomy, to treat uterine fibroids. This review highlighted that women in lower socioeconomic brackets tend to have poorer health outcomes and tend to not seek immediate care for uterine fibroids. Health literacy is linked to social determinants of health such as education and income. Incorporating interventions that focus on health literacy and education could drastically improve uterine fibroid treatment decisions. Community education regarding preventative care including regular pelvic exams may help ensure that all women, regardless of socioeconomic status, have early detection of uterine fibroids, thus potentially preventing the need for future hysterectomies. Providers should begin providing information on signs, symptoms, and risk factors for uterine fibroids so that women are aware of early signs. The information and education must be at an appropriate literacy level to ensure that patients understand signs and risks.

Additional research on provider-patient communication about uterine fibroid diagnosis and treatment is needed. Ensuring providers fully outline uterine fibroid diagnosis, as well as the risks and benefits of each treatment option is key to patient understanding. Providers must ensure that patients understand that treatment options vary based on each patient. These treatment options depend on a myriad of factors, including, but not limited to, severity of symptoms, number and location of fibroids, age and desire to have children. Ensuring that patients have an opportunity to ask questions and fully understand their uterine fibroid diagnosis and treatment options is vital to informed decision-making and an overall better quality of life.

While this review demonstrated that limited health literacy led to poor understanding of diagnosis and treatment, there is still a need for more research on the role of health literacy on uterine fibroid awareness and treatment options. Future research should correlate patients' health literacy levels through validated tools such as the Rapid Estimate of Adult Literacy in Medicine (REALM) or the Brief Health Literacy Screen (BHLS) and their understanding and treatment decisions.
